# Cloning, Expression and Functional Characterization of a Novel α-Humulene Synthase, Responsible for the Formation of Sesquiterpene in Agarwood Originating from *Aquilaria malaccensis*

**DOI:** 10.3390/cimb45110564

**Published:** 2023-11-10

**Authors:** Yasotha Sundaraj, Hasdianty Abdullah, Nima Ghahremani Nezhad, Afiq Adham Abd Rasib, Roohaida Othman, Kenneth Francis Rodrigues, Suriana Sabri, Syarul Nataqain Baharum

**Affiliations:** 1 Metabolomics Research Laboratory, Institute of Systems Biology (INBIOSIS), Universiti Kebangsaan Malaysia (UKM), Bangi 43600, Selangor, Malaysia; yasotha@unisel.edu.my; 2 Faculty of Engineering and Life Sciences, Universiti Selangor (UNISEL), Bestari Jaya 45600, Selangor, Malaysia; dianty@unisel.edu.my; 3 Department of Cell and Molecular Biology, Faculty of Biotechnology and Biomolecular Sciences, Universiti Putra Malaysia (UPM), Serdang 43400, Selangor, Malaysia; gs52916@student.upm.edu.my; 4 Department of Biological Sciences and Biotechnology, Faculty of Science and Technology, Universiti Kebangsaan Malaysia (UKM), Bangi 43600, Selangor, Malaysia; afiqadham.ar@gmail.com (A.A.A.R.); roohaida@ukm.edu.my (R.O.); 5 Biotechnology Research Institute, Universiti Malaysia Sabah (UMS), Kota Kinabalu 88400, Sabah, Malaysia; kennethr@ums.edu.my; 6 Enzyme and Microbial Technology Research Centre, Faculty of Biotechnology and Biomolecular Sciences, Universiti Putra Malaysia (UPM), Serdang 43400, Selangor, Malaysia; suriana@upm.edu.my

**Keywords:** α-humulene synthase, sesquiterpene, *Aquilaria malaccensis*, protein modeling, molecular docking

## Abstract

This study describes the cloning, expression and functional characterization of α-humulene synthase, responsible for the formation of the key aromatic compound α-humulene in agarwood originating from *Aquilaria malaccensis*. The partial sesquiterpene synthase gene from the transcriptome data of *A. malaccensis* was utilized for full-length gene isolation via a 3′ RACE PCR. The complete gene, denoted as *AmDG2*, has an open reading frame (ORF) of 1671 bp and encodes for a polypeptide of 556 amino acids. In silico analysis of the protein highlighted several conserved motifs typically found in terpene synthases such as Asp-rich substrate binding (DDxxD), metal-binding residues (NSE/DTE), and cytoplasmic ER retention (RxR) motifs at their respective sites. The *AmDG2* was successfully expressed in the *E. coli*:pET-28a(+) expression vector whereby an expected band of about 64 kDa in size was detected in the SDS-PAGE gel. In vitro enzyme assay using substrate farnesyl pyrophosphate (FPP) revealed that AmDG2 gave rise to two sesquiterpenes: α-humulene (major) and β-caryophyllene (minor), affirming its identity as α-humulene synthase. On the other hand, protein modeling performed using AlphaFold2 suggested that AmDG2 consists entirely of α-helices with short connecting loops and turns. Meanwhile, molecular docking via AutoDock Vina (Version 1.5.7) predicted that Asp307 and Asp311 act as catalytic residues in the α-humulene synthase. To our knowledge, this is the first comprehensive report on the cloning, expression and functional characterization of α-humulene synthase from agarwood originating from *A. malaccensis* species. These findings reveal a deeper understanding of the structure and functional properties of the α-humulene synthase and could be utilized for metabolic engineering work in the future.

## 1. Introduction

Aromas emitted by certain plants contain a mixture of hundreds of low-molecular-weight volatile compounds that differ between species, thus giving them their unique characteristic fragrances. For humans, fragrant plants, particularly flowers, constitute a commodity with strong aesthetic and emotional values [1]. In nature, these volatile compounds emitted by plants act as attractants in biotic interactions such as pollination [2] and seed dispersal [3], serve as direct or indirect defense against natural enemies [4] and play a role in protection against abiotic stresses such as water, light and temperature [5], which indirectly defends the respective plant from further damage. Plant volatiles are also of paramount importance in intra- and interspecific plant communication by alerting surrounding plants of potential pest/pathogen attacks via the transmission of airborne signals [6]. As a result, disease-resistant genes of the neighboring plants and in the healthy tissues of the infected plants will be activated.

Generally, plant volatiles fall into three main categories, namely the terpenoids (which is dominated by monoterpenoid and sesquiterpenoid), phenylpropanoids/benzenoids and fatty acid derivatives [7]. Of these, the terpenoid group has been extensively studied due to its vast application in the pharmaceutical and perfumery industries. Terpenoid is indeed a big family, comprising more than 55,000 compounds [8]. All terpenoids are derivatives from the common precursors isopentenyl diphosphate (IPP) and dimethylallyl diphosphate (DMAPP), which are synthesized through two independent pathways, namely the cytosolic mevalonate (MVA) pathway and the plastidic methylerythritol 4-phosphate (MEP) pathway [9]. Generally, the MVA pathway supplies the precursors for the production of sesquiterpenes (C_15_H_24_), while the MEP pathway, which primarily exists in eubacteria and plants, is responsible for producing hemiterpenes (C_5_H_8_), monoterpenes (C_10_H_16_), and diterpenes (C_20_H_32_). 

Sesquiterpenes represent the largest class of terpene compounds widely existing in plants, fungi, marine organisms, insects, and microbes. They have remarkable biological activities and have broad applications in food and flavor, fragrance and biofuels as well as in the pharmaceutical industry [10]. Many studies have been carried out on various plant species to clone and subsequently characterize a number of genes encoding the sesquiterpene enzymes that control the key steps in the secondary metabolic pathway [11].

*Aquilaria malaccensis* is a large evergreen tree that produces resin-impregnated wood (agarwood) that is fragrant and highly valuable throughout the globe, particularly across Asia and the Middle East. *A. malaccensis* is among the 13 agarwood-producing species recognized to date [12]. Agarwood is typically considered to be a pathological product produced by natural fungal invasion of the host [13], though many techniques are being employed nowadays to artificially induce its formation. Besides being used for flavor and in the perfumery industry, agarwood is also valued for its medicinal properties. It is generally agreed that a superior-quality agarwood essential oil possesses a minimum of 70% sesquiterpenoids, and the grading lowers based on the decrease in the percentage [14]. Although more than 210 sesquiterpenes have been identified from agarwood to date [15], the molecular mechanisms of sesquiterpene biosynthesis and regulation in agarwood remain to be fully understood. As such, this is a subject of interest for researchers worldwide, who are working extensively to decipher the underlying pathway. 

Many previous studies on terpene synthases in general and sesquiterpene synthases in particular have been reported in *A. sinensis*. Moreover, recent work has demonstrated that treated agarwood from *A. malaccensis* is rich in sesquiterpenes, with α-humulene being one of the key aromatic compounds in the essential oil [16]. Although α-humulene synthase (responsible for producing α-humulene) has been characterized in *A. crassna* [17], *A. sinensis* [15] and in a few other plants such as *Santalum austrocaledonicum* [18], *Picea glauca* [19], *Solanum habrochaites* [20], *Zingiber zerumbet* [21] and *Humulus lupulus* [22], to our knowledge, its isolation and characterization in *A. malaccensis* are still little understood. Hence, the objectives of this research are to clone, express and perform a thorough functional characterization of the enzyme α-humulene synthase from this plant species. 

The findings from this study provide novel insights into the structure and functions of the α-humulene synthase of *A. malaccensis* at the molecular level. Our investigations broaden the horizons for the bio-production of α-humulene, which is among the actively tapped fields of research under metabolic engineering. Sesquiterpene α-humulene is not only valued for its aroma but has also been reported to possess several medicinal properties as well [23,24,25]. Moreover, Alemdar et al. [26] have highlighted its usage as a precursor for the production of other medicinally important sesquiterpenes such as zerumbone, a potent anti-cancer agent. 

## 2. Materials and Methods

### 2.1. Plant Material

Plant material consisting of agarwood from a treated five-year-old *Aquilaria malaccensis* tree was collected from an experimental plot at Malaysia Nuclear Agency, Hulu Langat, Selangor (2.911567, 101.770096). The identification of *A. malaccensis* Lam. [27] was performed by A. Damanhuri (Curator for Universiti Kebangsaan Malaysia Herbarium; UKMB) for a voucher specimen named M. H. Azhari 1 [28]. The one-year-old treated sample was authenticated by Dr Chong Saw Peng, a researcher at the Malaysia Nuclear Agency. Holing of the tree trunk using a nail followed by inoculation with a mixture containing honey is the type of treatment given to induce agarwood formation in *A. malaccensis* trees [29]. The plant material was immediately frozen in liquid nitrogen and transferred to the Metabolomics Lab, Institute of Systems Biology (INBIOSIS), Universiti Kebangsaan Malaysia (UKM). There, the wood sample was cut into small pieces and dry-ground using a blender to obtain a fine fiber, which was stored at −80 °C before RNA extraction was carried out. 

### 2.2. Extraction of RNA and cDNA Synthesis

RNA extraction was undertaken using a FavorPrep™ Plant Total RNA Mini Kit for Woody Plants (Favorgen Biotech Corp, Ping Tung, Taiwan) following the manufacturers’ recommendations. Agarose gel electrophoresis and spectrophotometer analysis were conducted to determine the quality and quantity of the extracted RNA. Subsequently, it was reverse transcribed into 3′-Rapid Amplification of cDNA Ends (RACE)-Ready cDNA using SMARTer^®^ RACE cDNA Amplification Kit (Clontech, Mountain View, CA, USA) and diluted in 20 μL of Tricine-EDTA Buffer prior to storage at −20 °C.

### 2.3. Identification of Sesquiterpene Synthase Gene from A. malaccensis and Isolation of Its Full-Length Sequence

A partial sequence of the putative sesquiterpene synthase gene was obtained from the agarwood transcriptome set following data mining [30]. Several fundamental bioinformatics tools such as BLASTX (National Center for Biotechnology Information; NCBI), multiple sequence alignment using ClustalW (Kyoto University Bioinformatics Center) and open reading frame (ORF) finder (Swiss Institute of Bioinformatics; SIB) were employed to analyze and subsequently verify the identity of the gene. Following that, Primer 6 software was utilized to design the cDNA-gene-specific primer denoted as AmDG2 3′RACE_F (5′-GCGTCTGTGTTTGATGATACCTATGACA-3′) in order to isolate the full-length sequence of the truncated gene via a 3′-RACE PCR according to the manufacturer’s recommendation in the SMARTer^TM^ RACE cDNA Amplification Kit (Clontech, Mountain View, CA, USA). 

### 2.4. Ligation and Transformation of the Recombinant Vector into Host Cell

The obtained DNA fragment was ligated into pEASY^®^-T5 Zero Cloning Vector (TransGen, Beijing, China) and transformed into Trans1-T1 Phage Resistant Chemically Competent Cell using a heat shock method. Selection of positive transformant was carried out on an LB agar plate supplemented with 25 µg/mL of kanamycin and 100 µg/mL of ampicillin. Plasmid extraction from the positive transformant was performed using a Wizard^®^ Plus SV Minipreps DNA Purification System according to the manufacturer’s protocol (Promega, Madison, WI, USA), and the presence of the insert DNA was confirmed by PCR prior to sending for sequencing at First BASE Laboratories, Seri Kembangan, Selangor, Malaysia.

### 2.5. In Silico Analysis of the Sesquiterpene Synthase Gene 

Comparative sequence analysis of the desired gene was performed using several bioinformatics tools as aforementioned. The presence of signal peptide and its targeting location for the deduced proteins was predicted using SignalP 4.1 software [31] and TargetP-2.0 Server (DTU Health Tech). Meanwhile, the theoretical isoelectric point (pI) and predicted molecular weight (MW) of the proteins were determined using ExPASy Proteomic tools (Swiss Institute of Bioinformatics; SIB). The prediction of the protein structure was performed based on the structure of the 5-EPI-Aristolochene Synthase (5eat.1.A) as a template with a 43.58% sequence identity using AlphaFold2 (https://colab.research.google.com/github/sokrypton/ColabFold/blob/main/AlphaFold2.ipynb, accessed on 10 August 2023). The predicted model quality check was evaluated using two validation methods, VERIFY3D and Ramachandran Plots. To further evaluate the interaction between the target enzyme with its substrate, molecular docking analysis was performed. The SDF format of farnesyl pyrophosphate with a CID number of 445713 was downloaded from PubChem and converted to a PDB file through PyMol (Version 2.5.2). Energy minimization of ligand and protein was carried out using YASARA (version 17.4.17) through 5000 steps of the steepest descent method.

The nine poses were obtained through site-specific molecular docking using AutoDock Vina (Version 1.5.7) [32], and the protein molecule was initially prepared in AutoDock Vina by removing molecules of water, followed by the addition of polar hydrogen atoms and computing Gasteiger charges from the target protein. The x-, y-, and z- coordinates of the grid box center (within the active center) were set to 16.095, −5.042, and −5.983, respectively, and the size of the grid box was set to 50, 50, and 50 with a default grid point spacing of 0.375 Å.

### 2.6. Gene Synthesis and Expression of Sesquiterpene Synthase in E. coli

The full-length sesquiterpene synthase gene (denoted as *AmDG2*) was flanked with *Bam*HI and *Xho*I digestion sites and synthesized by service (GenScript, Piscataway, NJ, USA) following codon optimization. The synthesized gene was cloned into pET-28a(+) expression vector and transformed into *E. coli* BL21(DE3) (New England Biolabs, Frankfurt am Main, Germany) for the assessment of its enzyme expression. Meanwhile, the *E. coli* BL21(DE3) strain harboring empty pET-28a(+) vector was used as the control strain.

A single colony of recombinant *E. coli* BL21(DE3) harboring pET-28a(+):*AmDG2* was inoculated into 5 mL of LB medium containing kanamycin (25 µg/mL) and was grown overnight at 37 °C. The following day, the content was emptied into 45 mL of LB broth supplemented with 5 µL of kanamycin (25 µg/mL). The culture was induced with 1.0 mM IPTG at OD600 ~0.5. The cultures were incubated at 18 °C for 16 h with shaking at 250 rpm and were then harvested by centrifugation at 4000 rpm for 30 min at 4 °C. Subsequently, the bacteria was resuspended in 5 mL of 25 mM sodium phosphate buffer, pH 7.5, containing 0.5 M Tris–HCl, 5% glycerol, 1 mM dithiothreitol (DTT), 10 mM MgCl_2_, 1 mM MnCl_2_, pH 7.5, 1 mM lysozyme and 4 µL of β-mercaptoethanol (Sigma-Aldrich, St. Louis, MI, USA). The mixture was incubated on ice for 1 h and sonicated five times for 30 s using the Sonic Dismembrator Model 100 (Fisher Scientific, Hampton, NH, USA). The supernatant and cell pellet of the sonicated samples were separated using centrifugation at 4000 rpm for 30 min at 4 °C and finally stored separately at −20 °C until further use.

### 2.7. Enzyme Assay and Identification of Sesquiterpene Using GC-MS Analysis

The enzyme reaction was carried out as recommended by Kumeta and Ito [33] in a 4 mL vial with a solid-top polypropylene cap using 100 μL of crude protein extract in a final volume of 200 μL containing Tris-HCl buffer (25 mM, pH 7.0) supplemented with 10% glycerol, 100 mM MgSO_4_, 5 mM DTT, and 46 mM of (E,E)-farnesyl pyrophosphate (FPP) (Sigma-Aldrich, St. Louis, MI, USA). After incubation at 30 °C for 1 h, solid-phase microextraction (SPME) fiber was inserted into the headspace of the vial to collect volatiles for 1 min and then transferred to the injection port of a Clarus 600 GC-MS (PerkinElmer Inc., Waltham, MA, USA) that was equipped with a capillary column (Elite-5 30 m x 0.25 mm, film thickness 0.25 µm) with the initial temperature of the oven set to 250 °C for desorption of the sample. The flow rate of helium was at 1 mL min^−1^ at a constant pressure of 90 KPa. The program had an initial oven temperature of 80 °C followed by an increase of 5 °C min^−1^ until 220 °C with a 10-minute hold and then a further increase of 10 °C min^−1^ until 240 °C followed by a 3-minute final hold [33]. A full scan of the acquisition parameters includes a scan range from 40 to 350 amu. The identification of volatile compounds was based on the hit in NIST (version 2.0) and Wiley Registry 8th edition database of chemical library software. 

## 3. Results and Discussion

### 3.1. Full-Length Sesquiterpene Synthase (AmDG2) Sequence Analysis

The initial candidate sesquiterpene synthase gene (denoted as *AmDG2*) obtained from transcriptome data was a partial length of 948 bp and had the highest hit with delta-guaiene synthase from *Aquilaria sinensis* (55%). Following a 3′ RACE PCR, an approximately 1000 bp-sized fragment was obtained, successfully cloned and sequenced. The full-length *AmDG2* (GenBank accession No: OQ561767), 1671 bp in size, was compared using the BLASTX algorithm against the NCBI non-redundant protein database and showed a high amino acid sequence identity of 52–95% to terpene synthases of other *Aquilaria* members, as depicted in Table 1. 

**Figure 1:**
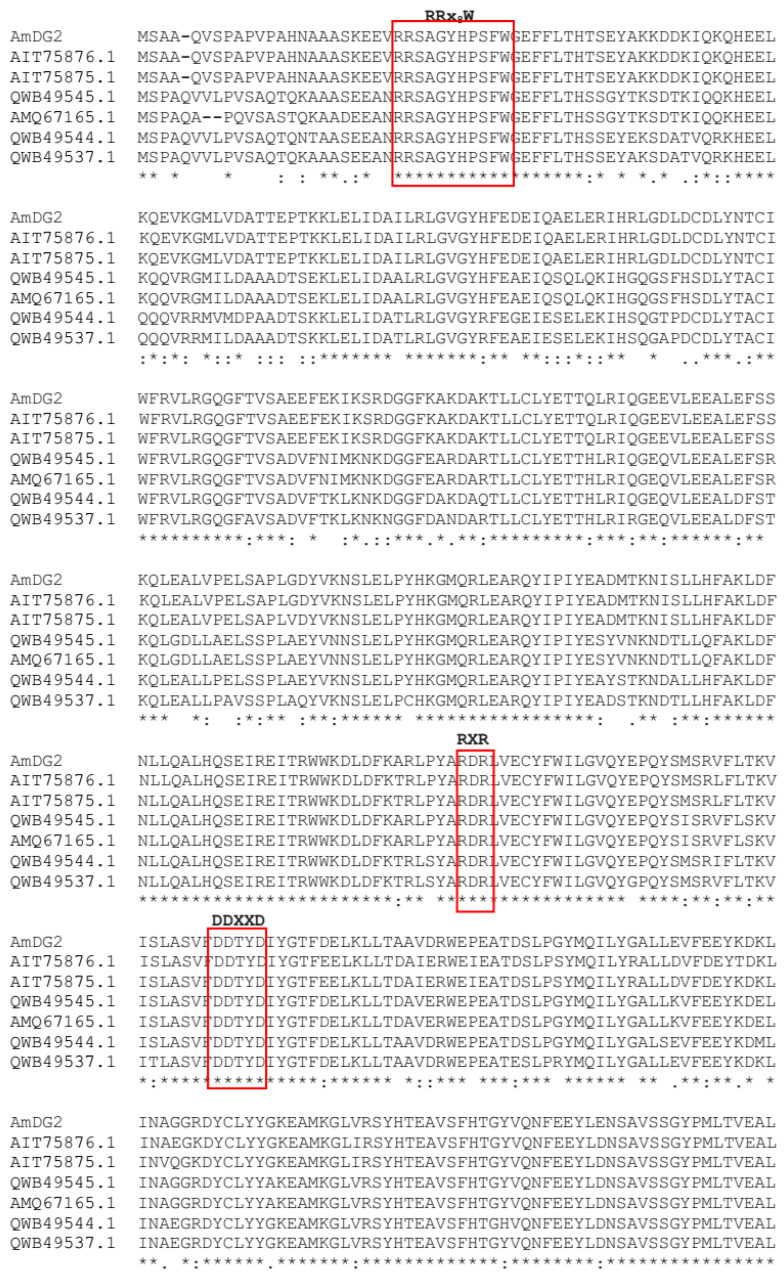

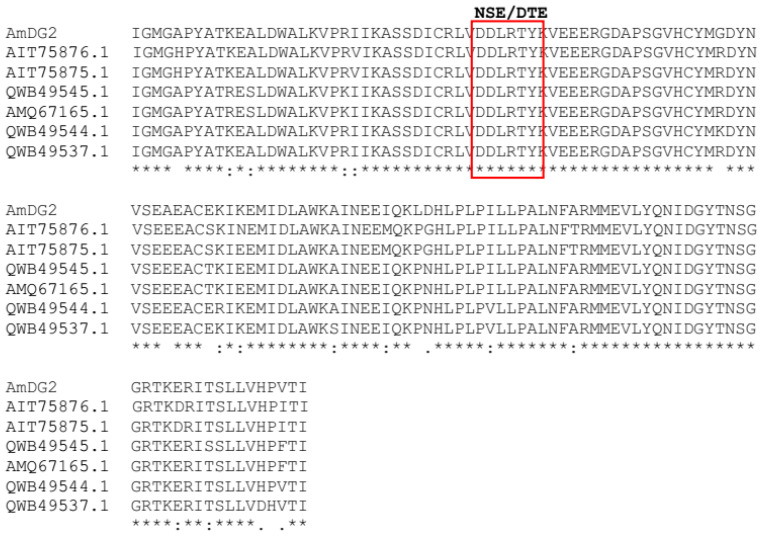
Multiple alignment of the full-length amino acid sequence of AmDG2 with sesquiterpene synthase from *A. sinensis* (AIT75876.1), putative delta guaiene synthase of *A. sinensis* (AIT75875.1), terpene synthase 11 from *A. sinensis* (QWB49545.1), alpha-humulene synthase of *A. crassna* (AMQ67165.1), terpene synthase 10 from *A. sinensis* (QWB49544.1) and terpene synthase 3 from *A. sinensis* (QWB49537.1). Amino acid residues conserved in all the sequences are marked with an asterisk (*). Conservation between amino acid groups of strongly similar properties are indicated by a colon (:) and conservation between amino acid groups of weakly similar properties are represented in period (.). The highly conserved RRx_8_W, RxR, DDxxD and NSE/DTE domains are highlighted in red boxes.

Multiple alignment of AmDG2 with other terpene synthases indicates highly conserved regions with all the terpene synthase signature motifs falling at their respective locations (Figure 1). Amongst them, the tandem arginine motif (RRx_8_W) located at the N-terminal region of terpene synthase has been reported to stabilize the closed active site in the enzyme–ligand complex reaction [34]. As reported by Jiang et al. [35], this motif serves an essential role in the catalysis of monoterpene cyclization. Wiliams et al. [36] determined that the deletion of this motif from *Mentha spicata* limonene synthase, a type of monoterpene synthase, affects its ability to utilize geranyl pyrophosphate as a substrate, suggesting that this motif might be involved in the isomerization of geranyl pyrophosphate to a cyclizable intermediate. On the other hand, the two highly conserved motifs of aspartate-rich substrate binding (DDxxD) and metal binding residues (NSE/DTE) that reside at the entrance to the active site coordinate the catalytic cycle of sesquiterpene synthase, which begins with binding of the diphosphate moiety of farnesyl pyrophosphate (FPP) to positively charged residues and Mg^2+^ cofactors [37]. Meanwhile, the arginine-rich RxR motif, which is located at 34 amino acids upstream of aspartate-rich motifs (DDxxD), is involved in the complexing of the diphosphate group, after the ionization of FPP [38,39]. Similar findings of these specific motifs were reported by Rusdi et al. [11] and Hattan et al. [8] in the sesquiterpene synthase genes from *Polygonum minus* and *Camellia hiemalis*, respectively.

Further analysis of the *AmDG2* that encodes for 556 amino acids revealed the calculated molecular mass as 63.6 kDa with an isoelectric point (pI) of 5.11 (ExPASy, Swiss Institute of Bioinformatics, SIB). The predicted amino acid sequence of AmDG2 was consistent with those of other plant sesquiterpene synthases encoding proteins of 550–580 amino acids, with molecular weights of 60–70 kDa [11]. A ProtParam analysis of the predicted amino acid sequence of AmDG2 revealed 88 negatively charged residues (Asp and Glu) and 63 positively charged residues (Arg and Lys), which represented the aliphatic index of this protein. SignalP-4.1 [31] and TargetP 2.0 prediction analysis, on the other hand, showed no signal peptide on the transmembrane regions, which is consistent with other sesquiterpene synthases in the range of 550–580 amino acids. The N-terminal transit peptide has been proposed to be necessary for plastidial targeting of hemi-, mono- and di- terpenes [40]. The absence of this N-terminal plastid targeting signal peptide suggests that AmDG2, in this study, is localized to the cytosol where FPP is found and where sesquiterpene biosynthesis takes place [41]. Besides the sequence similarity and identification of conserved motifs in their respective places, the absence of N-terminal plastid targeting signal peptide is noteworthy and further strengthens the fact that the designated AmDG2 is indeed a member of the sesquiterpene synthase family.

### 3.2. Structure Prediction, Validation and Site-Specific Molecular Docking

**Figure 2:**
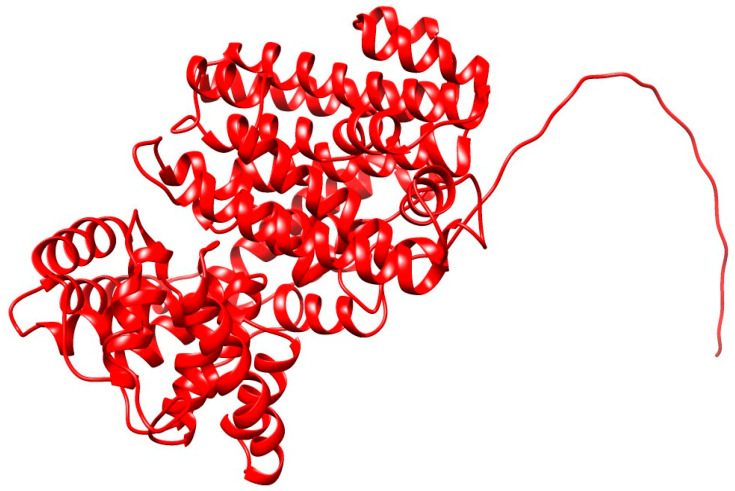
Three-dimensional protein model of AmDG2 predicted by AlphaFold2, which demonstrates the protein structure consisting of α-helices, short connecting loops and turns.

In this study, AlphaFold2, a groundbreaking tool for protein structure prediction, was employed to determine the structure of AmDG2, the results of which are outlined in Figure 2. AlphaFold2 marks a pivotal leap in protein structure prediction, enhancing the precision of structural predictions. This advance enables profound insights into the functional and dysfunctional aspects of proteins, particularly when there is a lack of experimentally determined homologous proteins [42]. 

**Figure 3:**
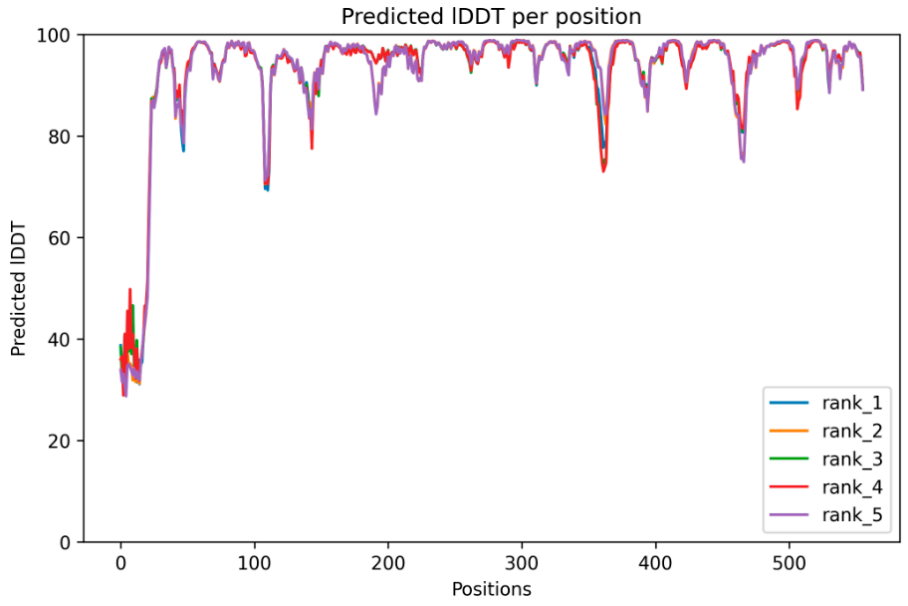
The top 5 models were predicted by AlphaFold2 and, among them, the model with the rank 1 (best model) showed the highest pLDDT score (93). Predicted models with ranks 1, 2, 3, 4, and 5 are shown in blue, orange, green, red, and violet, respectively.

The protein modeling has successfully predicted the protein model for the targeted AmDG2 with 43.58% identity with its template, 5-EPI-Aristolochene synthase (5eat.1.A). The predicted protein model revealed that AmDG2 consists entirely of α-helices and short connecting loops and turns (Figure 2). The results demonstrated that the rank 1 predicted model has the best predicted Local Distance Difference Test (pLDDT) score with the value of 93, where the values higher than 90 are strongly acceptable. AlphaFold2 produces three confidence metrics to gauge the precision of protein models. These metrics include the pLDDT, predicted Template Modeling Score (pTM), and Predicted Aligned Error (PAE). pLDDT is particularly noteworthy as it assigns confidence values to individual residues, ranging from 0 to 100, with higher values signifying greater confidence. Additionally, pLDDT serves as a dependable indicator of disorder content within the protein structure, making it a valuable tool for assessing both overall model accuracy and structural order [43]. Residues lower than 50 represent the unstructured part. As illustrated in Figure 2, the N-terminal residues from 1 to 25 are unstructured based on the pLDDT analysis that is also shown in the structure as a tail. The result of pLDDT analysis with a score of 93 is represented in Figure 3. The pLDDT score (0–100) is a per-residue confidence score, with values greater than 90 indicating high confidence and values below 50 indicating low confidence that can be attributed to the unstructured residues. 

**Figure 4:**
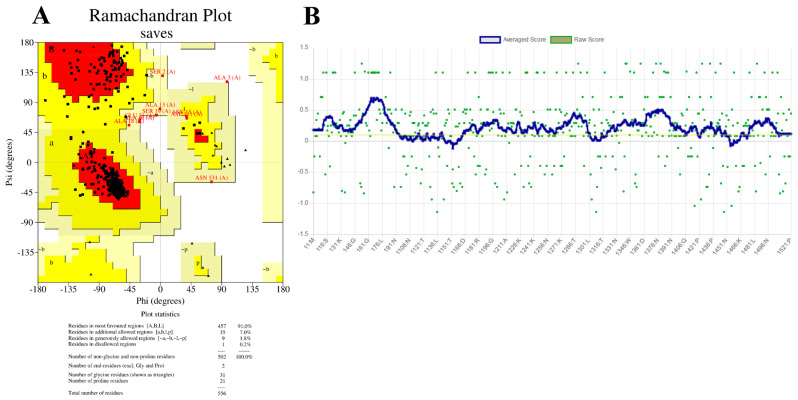
(**A**) Quality check on the AmDG2 protein model performed based on PROCHECK showing that 91% of the residues are in the most favored regions, indicating a good quality model based on the Ramachandran plot. (**B**) Measurement of the compatibility of the 3D structures using VERIFY3D, where 86.87% of the residues have averaged 3D-1D score ≥ 0.1.

Furthermore, the quality of the model was evaluated and validated by VERIFY3D and Ramachandran plots. The VERIFY3D result shown in Figure 4A illustrated that 86.87% of the residues have an average 3D-1D score ≥ 0.1, where a value of more than 80% is an acceptable score. In the meantime, another structure quality check performed based on Ramachandran plot analysis [44], as shown in Figure 4B, showed that 91% and 7% of residues are distributed in the most favored and additional allowed regions, respectively, which are acceptable results for a good protein model. Ramachandran plotting involves plotting the torsion angle φ values on the *x*-axis and the ψ values on the *y*-axis to predict the possible conformation of the peptide [45]. In computing a Ramachandran plot, atoms are treated as hard spheres whose dimensions correspond to their van der Waals radii. Any angle that results in the collision of the spheres is regarded as sterically unfavorable; hence, such conformations are also sterically not allowed. 

**Figure 5:**
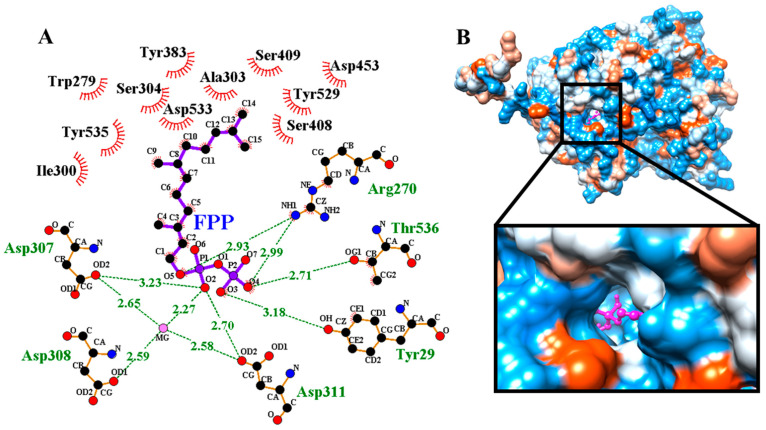
Site-specific molecular docking between the α-humulene synthase and FPP. (**A**) Analysis of hydrogen bonds between the enzyme and substrate, which have been illustrated with green dashes. The residues Asp307, Asp308, and Asp311 formed three hydrogen bonds towards Mg metal ion (shown as pink sphere). It is most likely that residues Asp307 and Asp311 act as catalytic residues. The presence of a Mg metal ion appears to be essential for the catalytic activity of this enzyme. The hydrophobic residues that are involved in the hydrophobic interactions are shown in dash semicircles. (**B**) The 3D localization of FPP inside the catalytic pocket is shown; the FPP is illustrated in magenta. The color of the predicted structure is based on the hydrophobicity of the surface.

Molecular docking results (Figure 5) illustrated that pose 1 has the highest binding affinity (least binding energies) of −8.1 (kcal/mol). The 2D analysis of molecular docking through LigPlot illustrated that residues Tyr209, Arg270, Asp307, Asp308, Asp31, and Thr536 are involved in the localization of the substrate FPP in the catalytic groove of α-humulene synthase through the formation of hydrogen bonds. Furthermore, the residues Trp279, Ile300 Ala303, Ser304, Ty383, Ser408, Ser409, Asp453, Tyr529, Asp533, and Tyr535 are involved in hydrophobic interactions between the α-humulene synthase and FPP (Figure 5A). The Asp-rich substrate binding conserved motif 307-DDTYD-311 plays the most important role in substrate binding through the formation of a hydrogen bond network between Asp307, Asp308, and Asp311 with a Mg metal ion and O_2_ atom of P_1_ located on the C_1_ of the FPP. This is similar to the studies reported by Harris et al. [46] and Ran et al. [15]. As such, it seems that Asp307 and Asp311 act as catalytic residues in the α-humulene synthase. Meanwhile, the 3D localization shown in Figure 5B demonstrates FPP inside the catalytic pocket, as illustrated in the color magenta. 

### 3.3. Expression of AmDG2 in E. coli

Following the heterologous expression of the sesquiterpene synthase in *E. coli*, the extracted crude protein was examined on an SDS-PAGE gel (Figure S1). The desired protein of approximately 64 kDa in size was successfully expressed in the *E. coli* BL21 (DE3) host cell, though the expression appears to be higher in the insoluble phase as compared to the soluble phase. Western blot analysis too confirmed the finding (Figure S2). Several factors could serve as the reason for obtaining the expressed protein in the soluble phase (cell pellet). Among these include the concentration of IPTG and even the culture condition applied (the temperature and induction time). It was noted that reducing the induction temperature improved recombinant protein folding and solubility by preventing its aggregation into the insoluble or cell pellet fraction [39,47,48,49,50,51]. Although induction was performed at a low temperature of 18 °C in this study, the desired protein was found to be overexpressed in the insoluble phase. Likewise, Haridhasapavalan et al. [52] also reported that induction at 18 °C did not favor any soluble expression of MESP1-NTH, but a faint expression was obtained at 30 °C for this construct. Similarly, Gutiérrez-González et al. [53] pinpointed that attempts at protocol optimization, including changes in IPTG concentration, culture temperature and even induction time, to obtain soluble proteins in their study were unsuccessful.

### 3.4. Enzyme Assay

**Figure 6:**
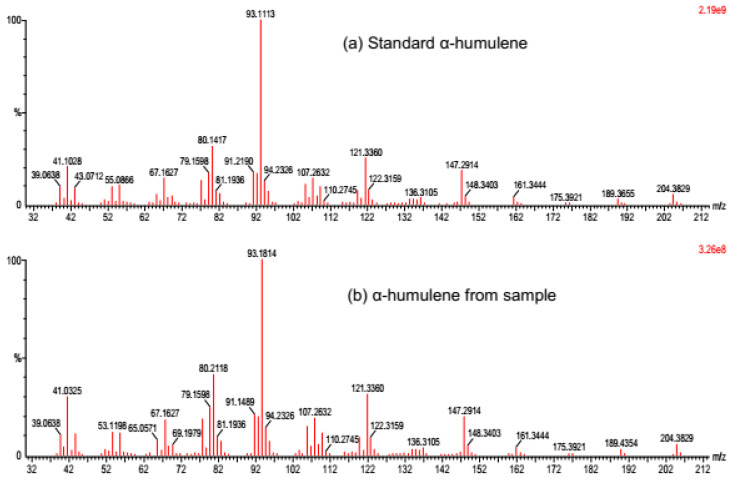
Fractionation spectrum of (**a**) standard α-humulene (Sigma-Aldrich, St. Louis, MI, USA) and (**b**) α-humulene from the sample. An identical fractionation pattern was observed in both spectra, thus verifying the compound as α-humulene.

In vitro enzymatic assay of the sesquiterpene synthase AmDG2 produced two different compounds, namely β-caryophyllene (at the minute of 11.65) and α-humulene (at the minute of 12.51), upon induction with substrate FPP (Figure S3). Of these two compounds, α-humulene, which is profusely present (almost 99%), contributes towards the agarwood fragrance, although β-caryophyllene, which was detected in trace amounts, was also detected in the essential oil of agarwood [16]. The result is in line with that of BLASTX analysis performed earlier on this gene, where *AmDG2* was reported to share high sequence identity with that of α-humulene synthase (85%) from *Aquilaria crassna*. Moreover, Alemdar et al. [54] demonstrated that α-humulene synthase from *Zingiber zerumbet* expressed in an *E. coli* BL21(DE3) strain converts substrate FDP to α-humulene (~94.5%) and β-caryophyllene (~5.5%), parallel to the results obtained in this study. Likewise, the fractionation spectrum of the detected compound α-humulene matches with that of the authentic standard (Sigma-Aldrich, USA), thus further confirming its identity as α-humulene synthase (Figure 6). Sesquiterpene humulene has been used widely in aromatherapy and has vast potential for medical applications due to its anti-inflammatory and anti-cancer activities [23,24].

## 4. Conclusions

In summary, α-humulene synthase from *A. malaccensis* was successfully cloned and characterized. The enzyme produced two industrially important sesquiterpenes: α-humulene (major) and β-caryophyllene (minor). It seems that Asp307 and Asp311 act as the catalytic residues in the α-humulene synthase. α-humulene is denoted as one of the key aromatic compounds in agarwood; as such, the identification of AmDG2 together with its active catalytic sites could be further utilized for metabolic engineering work in the future to increase the quantity and quality of production. Moreover, the current structural discovery of AmDG2 could facilitate future studies on the variations and specificities of terpene synthases in other plant species. 

## Figures and Tables

**Table 1:** BLASTX analysis of full-length *AmDG2* with the NCBI protein database.

Description	Organism	Score	E-Value	Identity (%)	Accession
Sesquiterpene synthase	*Aquilaria sinensis*	1048	0.0	94.84	AIT75876.1
Putative delta-guaiene synthase	*Aquilaria sinensis*	1046	0.0	94.66	AIT75875.1
Terpene synthase 10	*Aquilaria sinensis*	968	0.0	86.92	QWB49544.1
Terpene synthase 3	*Aquilaria sinensis*	953	0.0	85.82	QWB49537.1
Terpene synthase 11	*Aquilaria sinensis*	945	0.0	84.71	QWB49545.1
Alpha-humulene synthase	*Aquilaria crassna*	943	0.0	85.00	AMQ67165.1
Delta-guaiene synthase 4	*Aquilaria crassna*	626	0.0	52.40	AEG77020.1

## Data Availability

α-humulene synthase from *Aquilaria malaccensis* (GenBank accession No: OQ561767). Sesquiterpene synthase from *Aquilaria sinensis* (GenBank accession No: AIT75876.1). Putative delta-guaiene synthase from *Aquilaria sinensis* (GenBank accession No: AIT75875.1). Sesquiterpene synthase from *Aquilaria sinensis* (GenBank accession No: AIT75876.1). Terpene synthase 10 from *Aquilaria sinensis* (GenBank accession No: QWB49544.1). Terpene synthase 3 from *Aquilaria sinensis* (GenBank accession No: QWB49537.1). Terpene synthase 11 from *Aquilaria sinensis* (GenBank accession No: QWB49545.1). Alpha-humulene synthase from *Aquilaria crassna* (GenBank accession No: AMQ67165.1). Delta-guaiene synthase 4 from *Aquilaria crassna* (GenBank accession No: AEG77020.1).

## Supplementary Materials

The following supporting information can be downloaded at: https://www.mdpi.com/1467-3045/45/11/564/s1, Figure S1: SDS-PAGE gel of the expressed AmDG2 protein; Figure S2: Western Blot analysis of the expressed AmDG2 protein; Figure S3: GC-MS analysis for the in vitro enzymatic assay of AmDG2.
