# Correction: Sundaraj et al. Cloning, Expression and Functional Characterization of a Novel α-Humulene Synthase, Responsible for the Formation of Sesquiterpene in Agarwood Originating from *Aquilaria malaccensis*. *Curr. Issues Mol. Biol.* 2023, *45*, 8989–9002

**DOI:** 10.3390/cimb46070415

**Published:** 2024-07-04

**Authors:** Yasotha Sundaraj, Hasdianty Abdullah, Nima Ghahremani Nezhad, Afiq Adham Abd Rasib, Roohaida Othman, Kenneth Francis Rodrigues, Suriana Sabri, Syarul Nataqain Baharum

**Affiliations:** 1 Metabolomics Research Laboratory, Institute of Systems Biology (INBIOSIS), Universiti Kebangsaan Malaysia (UKM), Bangi 43600, Selangor, Malaysia; yasotha@unisel.edu.my; 2 Faculty of Engineering and Life Sciences, Universiti Selangor (UNISEL), Bestari Jaya 45600, Selangor, Malaysia; dianty@unisel.edu.my; 3 Department of Cell and Molecular Biology, Faculty of Biotechnology and Biomolecular Sciences, Universiti Putra Malaysia (UPM), Serdang 43400, Selangor, Malaysia; gs52916@student.upm.edu.my; 4 Department of Biological Sciences and Biotechnology, Faculty of Science and Technology, Universiti Kebangsaan Malaysia (UKM), Bangi 43600, Selangor, Malaysia; afiqadham.ar@gmail.com (A.A.A.R.); roohaida@ukm.edu.my (R.O.); 5 Biotechnology Research Institute, Universiti Malaysia Sabah (UMS), Kota Kinabalu 88400, Sabah, Malaysia; kennethr@ums.edu.my; 6 Enzyme and Microbial Technology Research Centre, Faculty of Biotechnology and Biomolecular Sciences, Universiti Putra Malaysia (UPM), Serdang 43400, Selangor, Malaysia; suriana@upm.edu.my

## Addition of Two Authors

Afiq Adham Abd Rasib and Roohaida Othman were not included as authors in the original publication [1]. The corrected Author Contributions statement appears here. The authors state that the scientific conclusions are unaffected. This correction was approved by the Academic Editor. The original publication has also been updated.

**Author Contributions:** Y.S. and S.N.B. conceptualized, designed the experiments and analyzed the data. Y.S. conducted the experiments and wrote the manuscript. H.A. and N.G.N. contributed to the protein modelling and molecular docking parts. S.S. and K.F.R. gave comments to improve the methods and validated the data analysis. A.A.A.R. and R.O. conducted the data mining analysis on the transcriptome data. All authors have read and agreed to the published version of the manuscript.
