# Supplementary material for: Cloning, Expression and Functional Characterization of a Novel α-Humulene Synthase, Responsible for the Formation of Sesquiterpene in Agarwood Originating from Aquilaria malaccensis
Source: Curr Issues Mol Biol. 2023 Nov 10;45(11):0. doi: 10.3390/cimb45110564 (PMC10670791; doi:10.3390/cimb45110564)
Supplement: Supplementary file 1 [file cimb-45-00564-s001.zip › Supplementary material Figure S1.pdf]

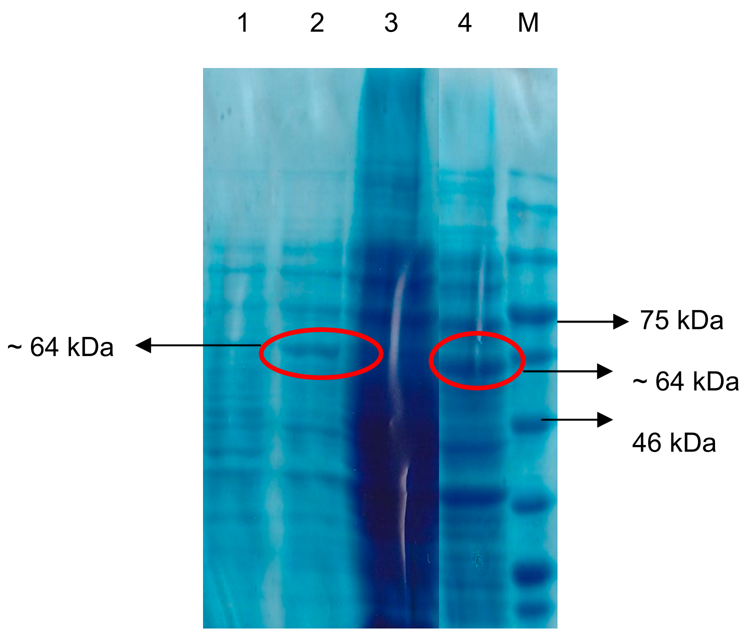

Figure S1. SDS-PAGE gel of the expressed AmDG2 protein. Lane 1 is the supernatant (soluble phase from uninduced *E.coli* BL21(DE3), lane 2 is the supernatant (soluble phase) from induced *E.coli* BL21(DE3), lane 3 is the cell pellet (insoluble phase) from uninduced *E.coli* BL21(DE3) and lane 4 is the cell pellet (insoluble phase) from induced *E.coli* BL21(DE3). Whereas lane M represent Blue Pre-stained Protein Standard (Broad range) from New England Biolabs, Frankfurt am Main, Germany. The red circle indicates the expressed protein in both the soluble and insoluble phases with lower expression detected in the former as compared to the latter.
