# Supplementary material for: Cloning, Expression and Functional Characterization of a Novel α-Humulene Synthase, Responsible for the Formation of Sesquiterpene in Agarwood Originating from Aquilaria malaccensis
Source: Curr Issues Mol Biol. 2023 Nov 10;45(11):0. doi: 10.3390/cimb45110564 (PMC10670791; doi:10.3390/cimb45110564)
Supplement: Supplementary file 1 [file cimb-45-00564-s001.zip › Supplementary material Figure S2.pdf]

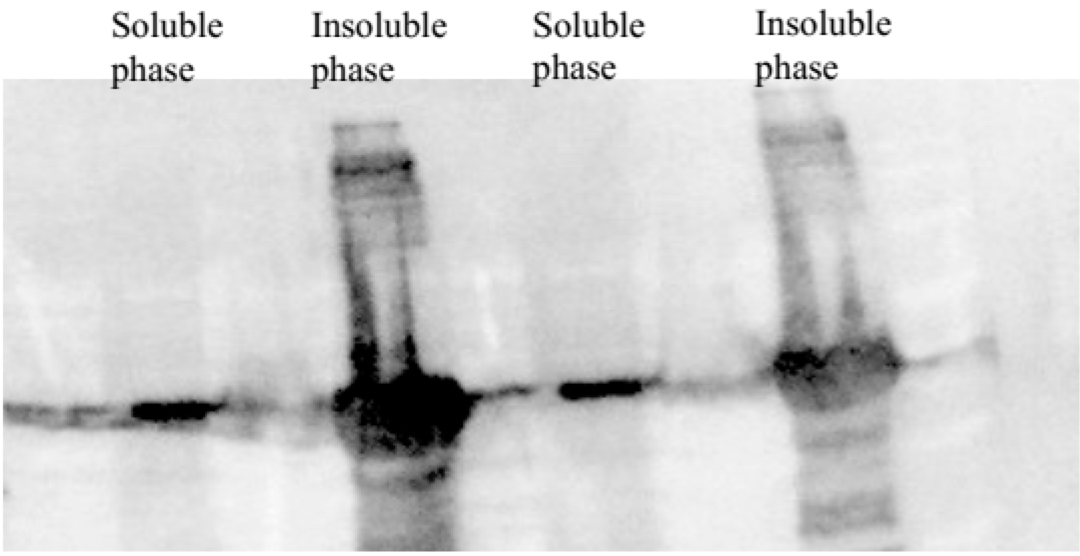


**Figure S2.** Western Blot analysis of the expressed AmDG2 protein. The expression in the insoluble phase is higher than the soluble phase.
